# Overnight switch from ropinirole to transdermal rotigotine patch in patients with Parkinson disease

**DOI:** 10.1186/1471-2377-11-100

**Published:** 2011-08-10

**Authors:** Han-Joon Kim, Beom S Jeon, Won Yong Lee, Myoung Chong Lee, Jae Woo Kim, Jong-Min Kim, Tae-Beom Ahn, Jinwhan Cho, Sun Ju Chung, Frank Grieger, John Whitesides, Babak Boroojerdi

**Affiliations:** 1 Departments of Neurology and Movement Disorder Center, Parkinson Disease Study Group, and Neuroscience Research Institute, BK21, College of Medicine, Seoul National University Hospital, Seoul, Korea; 2 Department of Neurology, Samsung Medical Center, Sungkyunkwan University School of Medicine, Seoul, Korea; 3 Parkinson/Alzheimer Center, Department of Neurology, Asan Medical Center, University of Ulsan College of Medicine, Seoul, Korea; 4 Department of Neurology, College of Medicine, Dong-A University, Busan, Korea; 5 Department of Neurology, Seoul National University Bundang Hospital, College of Medicine, Seoul National University, Seongnam, Korea; 6 Department of Neurology, BK21 and MRC, Kyung Hee University College of Medicine, Seoul, Korea; 7 UCB BioSciences GmbH, a member of the UCB Group of Companies, Monheim, Germany; 8 Schwarz BioSciences Inc, a member of the UCB Group of Companies, Raleigh, NC, USA

## Abstract

**Background:**

A recent trial involving predominantly Caucasian subjects with Parkinson Disease (PD) showed switching overnight from an oral dopaminergic agonist to the rotigotine patch was well tolerated without loss of efficacy. However, no such data have been generated for Korean patients.

**Methods:**

This open-label multicenter trial investigated PD patients whose symptoms were not satisfactorily controlled by ropinirole, at a total daily dose of 3 mg to 12 mg, taken as monotherapy or as an adjunct to levodopa. Switching treatment from oral ropinirole to transdermal rotigotine was carried out overnight, with a dosage ratio of 1.5:1. After a 28-day treatment period, the safety and tolerability of switching was evaluated. Due to the exploratory nature of this trial, the effects of rotigotine on motor and nonmotor symptoms of PD were analyzed in a descriptive manner.

**Results:**

Of the 116 subjects who received at least one treatment, 99 (85%) completed the 28-day trial period. Dose adjustments were required for 11 subjects who completed the treatment period. A total of 76 treatment-emergent adverse events (AEs) occurred in 45 subjects. No subject experienced a serious AE. Thirteen subjects discontinued rotigotine prematurely due to AEs. Efficacy results suggested improvements in both motor and nonmotor symptoms and quality of life after switching. Fifty-two subjects (46%) agreed that they preferred using the patch over oral medications, while 31 (28%) disagreed.

**Conclusions:**

Switching treatment overnight from oral ropinirole to transdermal rotigotine patch, using a dosage ratio of 1.5:1, was well tolerated in Korean patients with no loss of efficacy.

**Trial registration:**

This trial is registered with the ClincalTrails.gov Registry (NCT00593606).

## Background

Long term use of levodopa, the standard treatment for Parkinson's disease (PD), is associated with the development of motor complications, including motor fluctuations and dyskinesias [1]. Considerable evidence suggests that these motor symptoms are related to nonphysiological, pulsatile stimulation of dopamine receptors due to the intermittent administration of levodopa [2]. The longer half lives of dopamine agonists suggest they may provide more continuous and less pulsatile dopaminergic stimulation [3-5]. Their use can delay the initiation of levodopa treatment, a factor contributing to their widespread clinical acceptance. However, most currently available dopamine agonists require administration via multiple oral doses throughout the day, potentially leading to lower compliance and, albeit to a lesser degree than levodopa, motor fluctuations.

Rotigotine is a nonergolinic selective D_3_/D_2_/D_1 _dopaminergic agonist formulated in a silicone-based transdermal patch for once-daily application. It is released continuously over a 24-hour period and may provide more stable plasma concentrations than orally administered drugs. Studies have demonstrated safety and efficacy of the rotigotine patch, both in patients with early and advanced PD [6-8]. A recent trial involving predominantly Caucasian subjects showed switching PD treatment overnight from an oral dopaminergic agonist to the rotigotine patch can be well tolerated without loss of efficacy in terms of the Unified Parkinson's Disease Rating Scale (UPDRS) scores [9]. Overnight switching offers advantages over slow conversion, as a slow down-titration of the current medication and a slow up-titration of another are time-consuming, and can be associated with worsening of PD. However, no such data for rotigotine have been generated for Korean patients.

In this trial, the safety and tolerability of an overnight switch from ropinirole to the rotigotine transdermal patch, and the effects on motor and nonmotor symptoms of PD in Korean patients were investigated.

## Methods

### Patients

This open-label, multicenter trial was conducted at 8 sites in Korea between July and December 2007. Eligible subjects were Korean, male or female, aged 18 years or older with a diagnosis of PD (Hoehn and Yahr Stage I-IV) not satisfactorily controlled by ropinirole at a total daily dose of 3-12 mg, taken either as monotherapy or as an adjunct to levodopa. If subjects were receiving additional PD medications, including levodopa, anticholinergics, selegiline, amantadine, or entacapone, the total daily doses were maintained at a stable dose for at least 28 days before the study baseline and for the duration of the trial. Subjects were excluded if they had atypical parkinsonism; dementia, active psychosis, or hallucinations; clinically significant cardiac, renal or hepatic diseases; significant skin hypersensitivity to adhesive or other transdermal medications; recent contact dermatitis; symptomatic orthostatic hypotension; corrected QT interval of 500 ms or greater on electrocardiogram at pre-treatment evaluation or baseline or were pregnant or nursing. Prohibited medications included recent use of α-methyldopa, metoclopramide, neuroleptics, monoamine oxidase A inhibitors, methylphenidate, or amphetamine. Patients with evidence of an impulse control disorder (ICD) according to the Modified Minnesota Impulsive Disorder Interview (mMIDI) [10] at pre-treatment evaluation were also excluded.

### Study design

This trial was conducted in accordance with the Declaration of Helsinki and Good Clinical Practice Guidelines. The trial protocol was approved by the institutional review board at each center involved. All subjects provided informed consent prior to enrolment and could withdraw at any time.

### Treatments

Baseline data (Day 0) were recorded following an up to 28-day pre-treatment observation period, during which patient eligibility was confirmed and ropinirole and all other PD medications were maintained at stable doses. Subjects were planned to take their final dose of ropinirole in the afternoon or evening, and to apply a rotigotine patch upon awakening the next morning (Day 1). Switching from oral ropinirole to rotigotine patch was proposed to be done according to a predetermined dosage scheme: 3 mg/day ropinirole to 2 mg/24 hr rotigotine, 6 mg/day to 4 mg/24 hr, 8 or 9 mg/day to 6 mg/24 hr, and 12 mg/day to 8 mg/24 hr [9,11]. Throughout the 28-day treatment period patches were to be worn continuously for 24 hours prior to removal, at which point a new patch was to be immediately applied at a different position on the skin. Rotigotine dose adjustment by 2 mg/24 hr per week was allowed to optimize dosing. Following the treatment period, subjects entered the de-escalation period for up to 6 days, followed by a 28-day safety follow-up period.

### Evaluations

Efficacy of rotigotine was assessed by change from baseline to the end of treatment (EOT) using the Unified Parkinson's Disease Rating Scale (UPDRS) parts I, II, III, and IV. Changes in sleep-related problems were measured by the modified Parkinson's Disease Sleep Scale (mPDSS) [12] and the Epworth Sleepiness Scale (ESS). Changes in nonmotor symptoms were measured by the Non-motor Symptom Assessment Scale (NMSS) [13]. The severity of illness and global improvements were determined by the Clinical Global Impression (CGI), Patient Global Impression (PGI), and Parkinson's Disease Questionnaire (PDQ-8), in addition to a patient treatment preference scale. The efficacy scale assessment was done by the patient's neurologist. A Full Analysis Set, including all subjects with at least one valid post-baseline efficacy assessment, was used for efficacy evaluations. Due to the exploratory nature of this trial, only descriptive analyses were performed.

Blood sampling to determine unconjugated and total rotigotine plasma concentration (total rotigotine as the sum of the parent compound and its conjugates) was done prior to patch removal at the EOT visit (Day 28). Samples were analyzed using a liquid chromatography-mass spectrometry or mass spectrometry method. Rotigotine is metabolized to a great extent. Rotigotine is metobolised by N-dealkylation as well as direct and secondary conjugation. The main metabolites are sulfates and glucuronide conjugates of the parent compound and of the N-desalkyl-metabolites, which are biologically inactive.

Safety measures at baseline and at EOT included analysis of adverse events (AEs) and clinical laboratory evaluations, physical and neurological examinations, monitoring of changes in vital signs, body weight, and electrocardiograms, assessment of patch application sites, and results of the mMIDI. Treatment-emergent AEs were defined as those events that started on or after the date of first dose of rotigotine, or pre-existing events whose severity worsened on or after that date. In application site assessment, all abnormal clinically significant observations, other than reddening limited to the patch area after removal of the patch, was recorded as AEs. All subjects who applied at least one rotigotine patch were included in the analysis of safety (safety set).

Tolerability of switching was assessed by the following: (1) total number of subjects completing the trial, with or without adjustments to their original rotigotine dose assignment, (2) total number of subjects who discontinued or had dose reductions during the 5 half-life overlap period (the time from day of first treatment with rotigotine through 5 half lives of ropinirole), (3) total number of subjects who discontinued or had dose reductions due to AEs with onset during the 5 half-life overlap period and (4) incidence rates of AEs prior to, during, and after the switch (5 half-life overlap period) to rotigotine

## Results

### Subjects and Treatment Regimens

Of the 124 subjects enrolled, 116 (94%) received at least one dose of rotigotine and 99/116 (85%) completed the 28-day treatment period. The clinical and demographic characteristics of the subjects are listed in Table 1. The doses of rotigotine initiated at baseline were 2 mg/24 hr in 26 subjects, 4 mg/24 hr in 30 subjects, 6 mg/24 hr in 31 subjects, and 8 mg/24 hr in 29 subjects.

**Table 1:** Baseline Characteristics (safety set)

	N = 116
Age, yrs ± SD	60.0 ± 10.1
Gender, male, n (%)	69 (59.5)
Body weight, kg ± SD	64.2 ± 9.5
BMI, kg/m^2 ^± SD	24.2 ± 2.9
Duration of PD, yrs ± SD	5.4 ± 4.0
H&Y stage, n (%)	
1	21 (18.1)
2	69 (59.5)
3	21 (18.1)
4	5 (4.3)
Concomitant PD medication, n (%)	
Levodopa	100 (86.2)
Amantadine	61 (52.6)
Selegiline	61 (52.6)

SD = Standard Deviation.

### Efficacy

Rotigotine treatment resulted in small mean decreases in UPDRS I, II, III, and IV (Table 2). Compared with baseline, mean UPDRS III score was reduced from 17.9 ± 10.3 (n = 114) to 15.9 ± 10.0 (n = 112).

**Table 2:** UPDRS scores and NMSS scores at baseline and end of treatment

	Baseline (n = 114)	End of Treatment (n = 112)	Difference (n = 112)
UPDRS			
I	1.9 ± 1.8	1.4 ± 1.6	-0.5 ± 1.2
II	7.8 ± 5.3	6.9 ± 4.9	-0.9 ± 3.3
III	17.9 ± 10.3	15.9 ± 10.0	-1.9 ± 5.9
IV	2.7 ± 3.1	2.2 ± 2.8	-0.4 ± 1.8

	**Baseline (n = 114)**	**End of Treatment (n = 111)**	**Difference (n = 111)**

NMSS			
Total	29.5 ± 30.5	21.8 ± 22.2	-7.9 ± 19.8
Cardiovascular	0.8 ± 1.3	0.7 ± 2.6	-0.0 ± 2.7
Sleep/fatigue	5.8 ± 7.3	4.8 ± 6.0	-0.9 ± 6.7
Mood/cognition	4.6 ± 9.1	2.7 ± 6.6	-1.9 ± 6.2
Perceptual problems	0.2 ± 1.3	0.1 ± 0.7	-0.1 ± 1.1
Attention/memory	3.4 ± 4.7	2.4 ± 4.1	-1.0 ± 3.7
Gastrointestinal	2.3 ± 4.0	1.7 ± 3.0	-0.6 ± 3.6
Urinary	6.3 ± 9.9	5.2 ± 7.6	-1.3 ± 5.3
Sexual dysfunction	1.3 ± 3.5	0.9 ± 3.2	-0.4 ± 1.8
Miscellaneous	4.8 ± 6.4	3.3 ± 5.4	-1.6 ± 5.6

UPDRS, Unified Parkinson's Disease Rating Scale; NMSS, Non-Motor Symptom Scale.

Plus-minus values are mean ± SD (Standard Deviation).

Measurements of sleep-related symptoms showed no substantial changes. The mean mPDSS score was 12.7 at baseline (n = 114), and the change from baseline to EOT was -0.8 (n = 112). The mean ESS score was 6.0 at baseline (n = 63) and 5.7 at EOT (n = 57). At baseline six subjects were defined as excessively sleepy (ESS ≥10), of whom five no longer experienced excessive somnolence at EOT. Three additional subjects, who were not considered excessively sleepy at baseline, were classified as excessively sleepy following treatment with rotigotine.

A reduction of 7.9 in the total NMSS score was observed at EOT (n = 111, Table 2). The baseline total NMSS score was 29.5 ± 30.5 (n = 114). Scores for each of the individual domains showed a reduction from baseline.

At EOT, CGI scores as assessed by investigators indicated that 54 subjects were considered improved, while 22 were considered worsened and 37 had no change (n = 114). In the remaining one patient, CGI score was not assessed. The mean CGI score for global improvement was 3.6, indicating an average assessment of minimally improved to no change. The PGI scores showed that 54 subjects reported improvement, while 27 subjects reported worsening (n = 114). As part of this rating, 77 subjects reported having no side effects, and 24 subjects reported side effects that 'did not significantly interfere' with their functioning. In five cases patients considered their side effects 'outweighed the therapeutic effect.'

The mean baseline PDQ-8 score was 22.3 (n = 114), and the change from baseline to EOT was -3.9 (n = 112), indicating some improvement in the occurrence of various problems associated with PD upon switching to rotigotine.

Patient treatment preference scale analysis at EOT showed that slightly less than half of the subjects (52 of 114 subjects, 46%) agreed that they preferred using a patch over oral medications, while 31 (28%) disagreed and 29 (26%) neither agreed nor disagreed. The proportions of subjects who were either 'satisfied' or 'very satisfied' were 37% with rotigotine and 44% with ropinirole. The aspects of the patch that subjects liked the most were once-a-day application (72%), non-interference with normal activities (54%), and not having to remember to take medicine during the day (54%). The aspect that subjects liked the least was the patch not staying on for the entire day (71%). As determined by the investigator at EOT, patch adhesiveness for the last patch administered was < 50% in three of 73 subjects assessed; in two of these subjects the patch was "detached".

### Safety and Tolerability

Of the 99 subjects who switched from ropinirole and completed the treatment period, most (n = 88) did not require rotigotine dose adjustment. Of the 11 who did require dose adjustment 10 required a dose increase, and 1 required a decrease followed by an increase to the initial dose.

A total of 76 treatment-emergent AEs were reported in 45 subjects (39%), none of which were considered serious. The most commonly reported AEs were dizziness and tremor (Table 3). AEs were generally mild or moderate in intensity, with a severe AE reported by only one subject, who experienced a severe dyskinesia after 15 days of treatment. The dyskinesia resolved the next day without a change in rotigotine dose. The incidence rate of AEs was highest during the overlap period (0.112 AEs per day) compared with prior to (0.004) or after the 5 half-life overlap period (0.008). Sixteen subjects experienced a total of 26 AEs that started during the overlap period, of which the most common were somnolence, dizziness, dyskinesia, and tremor, reported by three subjects each. No subject developed impulse control disorder during the trial.

**Table 3:** The most common treatment-emergent adverse events with an incidence of 2% or greater

Treatment-emergent adverse event	Incidence, n (%)
Dizziness	7 (6.0)
Tremor	7 (6.0)
Dyskinesia	6 (5.2)
Bradykinesia	4 (3.4)
Somnolence	4 (3.4)
Muscle Rigidity	5 (4.3)
Asthenia	6 (5.2)
Dry mouth	4 (3.4)
Application and Instillation Site Reactions*/Application site pruritis	3 (2.6)

*Application and Instillation Site Reactions(MedDRA high-level term, MedDRA version 9.1) comprising erythema, pruritus, rash, irritation, eczema, vesicles, inflammation, and other reactions. Application site pruritus was the only Preferred Term for this High Level Term.

The total number of subjects who discontinued due to AEs with onset during the 5 half-life overlap period was 9(7.8%) and the total number of subjects who had dose reductions due to AEs with onset during the 5 half-life overlap period was 1(0.9%). The total number of subjects who discontinued during the 5 half-life overlap period (the time from day of first treatment with rotigotine through 5 half lives of ropinirole) was 0(0%) and the total number of subjects who had dose reductions during the 5 half life overlap period (the time from day of first treatment with rotigotine through 5 half lives of ropinirole) was 1(0.9%). AEs that led to discontinuation of more than one subject were tremor and dizziness (three each), and aggravated parkinsonism and dyskinesia (two each). However, no AE that led to discontinuation was severe in intensity and all were resolved by EOT.

In skin assessment, eleven subjects experienced minimal and barely perceptible erythema and three experienced definite erythema with minimal edema limited to the patch area at application sites. Itching at the application site developed in 17 subjects and was considered clinically relevant in three subjects. Because only clinically significant observations were counted as AEs in skin assessment (see Methods), only these three subjects were listed in Table 3. One of these three subjects withdrew from the trial prematurely due to itching at the application site.

### Pharmacokinetics

Mean unconjugated and total rotigotine plasma concentrations under steady-state conditions in subjects increased in a dose-proportional manner (Table 4). The ratio of total rotigotine to unconjugated rotigotine was similar at all doses evaluated.

**Table 4:** Rotigotine plasma concentrations at end of treatment

Daily rotigotine dose	n≧LOQ	ng/mL (unconjugated)	ng/mL (total)	Ratio (total rotigotine/ unconjugated rotigotine)
2 mg/24 hr	14	0.265 ± 0.146	1.995 ± 0.736	8.447 ± 2.919
4 mg/24 hr	24	0.559 ± 0.236	4.157 ± 1.534	7.922 ± 2.753
6 mg/24 hr	27	0.928 ± 1.005	6.620 ± 3.962	8.802 ± 4.871
8 mg/24 hr	23	1.215 ± 0.790	7.433 ± 4.423	6.968 ± 3.071

Plus-minus values are mean ± SD (Standard Deviation).

LOQ = limit of quantification (0.01 ng/mL for unconjugated Rotigotine, 0.01 ng/mL for total Rotigotine)

Note: Values below LOQ were replaced by 0 in calculations (Mean, SD, CV, Med, Min, Max)

## Discussion

This open-label trial showed that switching dopaminergic PD treatment overnight from oral ropinirole to transdermal rotigotine patch was generally well tolerated without loss of efficacy. Most AEs were consistent with stimulation of dopaminergic receptors or the use of a transdermal patch, with an incidence that was lower than or comparable to previous studies with rotigotine patch [7,9]. This trial was not designed to make determinations regarding the efficacy of rotigotine and not powered to show superiority of one drug over the other. However, substituting rotigotine for ropinirole appeared to improve both motor and nonmotor symptoms of PD, as measured by reductions from baseline values in UPDRS, NMSS, mPDSS, and ESS scores. A randomized controlled trial has demonstrated significant benefits with rotigotine versus placebo in the control of motor function and nocturnal sleep disturbance in PD patients [14].

It should be noted that the observed improvements occurred with a dosage conversion rate of 1.5:1 for ropinirole to rotigotine. In a previous overnight switch study, higher doses of rotigotine were used, in a dosage conversion rate of 1:1, but the reduction in UPDRS III score after switch from ropinirole to rotigotine was smaller than in the current study [9]. The seemingly greater potency of rotigotine in this trial is not likely to be due to the differences in pharmacokinetics, as the plasma concentration of rotigotine was comparable to plasma concentrations obtained in other studies [15,16]. However, it may be attributable to differences in ethnicity, as previous studies enrolled predominantly Caucasian subjects, or the baseline severity of PD motor symptoms.

It is not clear whether the reductions in scores for nonmotor symptoms are related to the reduction in scores for severity of motor symptoms. Some studies have shown a correlation between motor and nonmotor symptoms [13,17,18]. However, the absence of a correlation has also been reported [19,20], and it is possible that the reduction in scores for nonmotor symptoms after switching reflects a difference between ropinirole and rotigotine's effects on nonmotor symptoms, independent of motor symptoms [21].

## Conclusions

This trial showed that overnight switch from oral ropinirole to transdermal rotigotine, with a dose conversion ratio of 1.5:1, was well tolerated in Korean patients with no apparent loss of efficacy. However, due to the open-label design and exploratory nature of this trial, further investigation will be required to ascertain the relative efficacy of the rotigotine patch following a switch from oral ropinirole.

## Competing interests

The authors declare that they have no competing interests.

## Financial Disclosure

Dr. HJ Kim reports no disclosures.

Dr. BS Jeon reports no disclosures.

Dr. WY Lee reports no disclosures.

Dr. MC Lee reports no disclosures.

Dr. JW Kim reports no disclosures.

Dr. JM Kim reports no disclosures.

Dr. TB Ahn reports no disclosures.

Dr. J Cho reports no disclosures.

Dr. SJ Chung reports no disclosures.

John Whitesides is a salaried employee of UCB and receives UCB stock options as an employee of UCB.

Babak Boroojerdi is a salaried employee of UCB and receives UCB stock options as an employee of UCB.

Frank Grieger is a salaried employee of UCB.

## Authors' contributions

HJK conceived of, organized, and executed the study, critically reviewed the statistical analysis, and wrote the first draft. BSJ conceived of, organized, and executed the study, critically reviewed the statistical analysis and manuscript. WYL executed the study, reviewed the statistical analysis, and critically reviewed the manuscript. MCL executed the study, reviewed the statistical analysis, and critically reviewed the manuscript. JWK executed the study, reviewed the statistical analysis, and critically reviewed the manuscript. JMK executed the study, reviewed the statistical analysis, and critically reviewed the manuscript. TBA executed the study, reviewed the statistical analysis, and critically reviewed the manuscript. JC executed the study, reviewed the statistical analysis, and critically reviewed the manuscript. FG organized the study, designed and executed the and critically reviewed the manuscript. SJC executed the study, reviewed the statistical analysis, statistical analysis, and critically reviewed the manuscript. JW organized the study, designed and reviewed the statistical analysis, and critically reviewed the manuscript. BB organized the study, designed and reviewed the statistical analysis, and critically reviewed the manuscript. All authors read and approved the final manuscript.

## Pre-publication history

The pre-publication history for this paper can be accessed here:

http://www.biomedcentral.com/1471-2377/11/100/prepub
